# Effects of motor imagery based brain-computer interface on upper limb function and attention in stroke patients with hemiplegia: a randomized controlled trial

**DOI:** 10.1186/s12883-023-03150-5

**Published:** 2023-03-31

**Authors:** Xiaolu Liu, Wendong Zhang, Weibo Li, Shaohua Zhang, Peiyuan Lv, Yu Yin

**Affiliations:** 1 College of Nursing and Rehabilitation, North China University of Science and Technology, Tangshan, 063210 China; 2 Department of Rehabilitation, Hebei General Hospital, Shijiazhuang, 050000 China; 3 Department of Gastrointestinal Surgery, The Second Hospital of Hebei Medical University, Shijiazhuang, 050000 China; 4 Department of Medical, The Eighth People’s Hospital of Hebei Province, Shijiazhuang, 050000 China; 5 Department of Neurology, Hebei General Hospital, Shijiazhuang, 050000 China; 6 Hebei Provincial Key Laboratory of Cerebral Networks and Cognitive Disorders, Shijiazhuang, 050000 China

**Keywords:** Brain-computer interface, Stroke, Attention, Motor imagery, Upper extremity

## Abstract

**Background:**

Seeking positive and comprehensive rehabilitation methods after stroke is an urgent problem to be solved, which is very important to improve the dysfunction of stroke. The aim of this study was to investigate the effects of motor imagery-based brain-computer interface training (MI-BCI) on upper limb function and attention in stroke patients with hemiplegia.

**Methods:**

Sixty stroke patients with impairment of upper extremity function and decreased attention were randomly assigned to the control group (CR group) or the experimental group (BCI group) in a 1:1 ratio. Patients in the CR group received conventional rehabilitation. Patients in the BCI group received 20 min of MI-BCI training five times a week for 3 weeks (15 sessions) in addition to conventional rehabilitation. The primary outcome measures were the changes in Fugl-Meyer Motor Function Assessment of Upper Extremities (FMA-UE) and Attention Network Test (ANT) from baseline to 3 weeks.

**Results:**

About 93% of the patients completed the allocated training. Compared with the CR group, among those in the BCI group, FMA-UE was increased by 8.0 points (95%CI, 5.0 to 10.0; *P* < 0.001). Alert network response time (32.4ms; 95%CI, 58.4 to 85.6; *P* < 0.001), orienting network response (5.6ms; 95%CI, 29.8 to 55.8; *P* = 0.010), and corrects number (8.0; 95%CI, 17.0 to 28.0; *P* < 0.001) also increased in the BCI group compared with the CR group. Additionally, the executive control network response time (− 105.9ms; 95%CI, − 68.3 to − 23.6; *P* = 0.002), the total average response time (− 244.8ms; 95%CI, − 155.8 to − 66.2; *P* = 0.002), and total time (− 122.0ms; 95%CI, − 80.0 to − 35.0; *P* = 0.001) were reduced in the BCI group compared with the CR group.

**Conclusion:**

MI-BCI combined with conventional rehabilitation training could better enhance upper limb motor function and attention in stroke patients. This training method may be feasible and suitable for individuals with stroke.

**Trial registration:**

: This study was registered in the Chinese Clinical Trial Registry with Portal Number ChiCTR2100050430(27/08/2021).

## Background

Stroke is the leading cause of disability worldwide [1]. In China, the burden of stroke disease has been steadily increasing on a yearly basis [2]. Moreover, up to 55–75% of stroke survivors have varying degrees of upper limb motor dysfunction, which not only seriously affects the patient’s independence in daily living activities but also brings a huge economic burden to families and society [3, 4]. Thus, improving the motor function of the limbs in these patients, especially the upper limbs, is of essential importance. However, this is a difficult and long-term rehabilitation [5].

In addition, attention disorders and slowing information processing speed are common in stroke patients [6], affecting the rehabilitation process and functional outcomes [7]. Attention impairment increases the time needed for recovery of motor function. Therefore, positive and comprehensive rehabilitation methods and accelerating the rehabilitation process for stroke patients are urgent problems that need to be solved.

Motor imagery (MI) without movement execution can promote the recovery of motor function after stroke [8]. Motor imagery, also known as the internal mental rehearsal of physical movement tasks and the accompanying experience of sensory information without a direct external stimulus, can activate sensorimotor areas similar to actual motion [9, 10]. Motor imagery-based brain-computer interface (MI-BCI), today widely used for rehabilitation of motor abilities and prosthesis control for patients with motor impairments [11], can achieve the effect of rehabilitation training by collecting the EEG signal of the patients’ motor imagination, using the computer to extract, decode, classify and identify the movements and accuracy of the patients’ motor imagination, and connecting external devices, such as functional electrical stimulator (FES) and exoskeleton robots to give feedback [12]. In their study, Ang et al. [13] found that more than 60% of stroke patients achieved significant improvements in motor function after 4 weeks of MI-BCI training. The remarkable improvement in motor function after stroke is based on neuroplasticity. Miao et al. [14] found that MI-BCI could effectively improve the upper limb function of stroke patients in the sequela stage after 4 weeks of MI-BCI training combined with virtual limbs and functional electrical stimulation (FES) as feedback. The central intervention effect of MI-BCI training on the cerebral cortex and the promotion of cortical remodeling were confirmed by a brain topographic map. In addition, because MI is usually concealed within patients, MI-BCI can also monitor the accuracy of patients’ motor imagery and provide feedback during training to help patients timely adjust their training status. Pichiorri et al. [10] recruited 28 subacute patients and studied the efficacy of motor imagery with or without BCI support, reporting a significant and clinically relevant motor functional recovery for the BCI group. In addition, using BCIs for cognitive training is another emerging topic in the field of neurorehabilitation. BCI has been used as a neurofeedback platform to effectively improve the attention level of patients with attention deficit hyperactivity disorder (ADHD) and stroke [12, 15]. In Toppi’s study [16], stroke patients were instructed to voluntarily increase their sensory-motor rhythm (SMR: 12–15 Hz) amplitude over an established threshold set by BCI. By analyzing the electroencephalogram data before and after training, it was confirmed that BCI-controlled neurofeedback intervention could improve cognitive function after stroke. Gonzales et al. [15] designed and developed a BCI system to evaluate and treat ADHD using θ/β as a parameter scheme to focus attention on cognitive tasks in combination with games.

American Stroke Association (ASA) recommends early rehabilitation treatment to the patients hospitalized for stroke and early rehabilitation can improve the rehabilitation effect and reduce other complications [17]. Indeed, most previous studies on the effectiveness of post-stroke upper limb rehabilitation interventions excluded individuals with cognitive deficits who suffered stroke [18]. On the other hand, relationships between cognitive and motor deficits are being increasingly identified. Rinne et al. [6] suggested that methods to improve attentional control may confer secondary benefits on physical rehabilitation. Daly et al. [19] found that short-term intensive MI training could strengthen attention, maintain high levels of alertness, and shorten exercise preparation time.

Despite promising results achieved so far, the application of MI-BCI in stroke rehabilitation is still in its early stages, and different clinical outcomes have been reported. Most studies on stroke patients are limited by small sample sizes, thus providing insufficient evidence to demonstrate the effectiveness of MI-BCI on upper limb function in stroke patients [4, 20]. Although BCI can be used to improve attention in people with attention deficit, the use of MI-BCI to improve attention has not been studied. Most of the previous MI-BCI studies focused on the motor function of stroke patients, ignoring that patients’ attention may be improved in the process of repetitive motor imagination and feedback to obtain better rehabilitation effects. Therefore, in this study, we conducted MI-BCI combined with conventional rehabilitation training for stroke patients for 3 weeks, and took the difference of clinical tests before and after intervention as the indicator to evaluate the effectiveness of the training, aiming to investigate if MI-BCI can better improve the upper limb function and attention in stroke patients on the basis of conventional rehabilitation. We also investigated whether there is a correlation between motor function recovery and attention improvement, so as to guide the development of a rich and personalized rehabilitation program.

## Methods

### Ethics statement

The purpose and requirements of the study were explained to all patients, and all patients signed informed consent before the start of the trial. The study followed the Declaration of Helsinki and Good Clinical Practice. Ethics committee approval was obtained from the Ethics Committee of the Hebei Provincial People’s Hospital (2021 Scientific Research Ethics Review [301]).

### Sample size calculation

The sample size was calculated according to the study’s primary outcome measure. We used the Fugl-Meyer results from Pichiorri’s study [10] and the attention network test results from LaCroix’s study [21]. Two-sample mean comparison estimation formula was as follows:

$${\text{n}} = \frac{{{{\left( {{Z_\alpha } + {Z_\beta }} \right)}^2}\left( {1 + {\raise0.7ex\hbox{$1$} \!\mathord{\left/{\vphantom {1 K}}\right.\kern-\nulldelimiterspace}\!\lower0.7ex\hbox{$K$}}} \right){\sigma ^2}}}{{{\delta ^2}}}$$

$$\delta = \left| {\overline {{X_e}} - \overline {{X_c}} } \right|$$

$${\varvec{S}}^{2}=\frac{({S}_{e}^{2}+{KS}_{c}^{2})}{(1+\raisebox{1ex}{$1$}\!\left/ \!\raisebox{-1ex}{$\varvec{K}$}\right.)}$$

where n is the number of patients in the experimental group or the control group, Z is the standard normal deviation boundary value; α = 0.05, β = 0.90, two-sided test, ${Z}_{\alpha }$=1.64, ${Z}_{\beta }$=1.28; $\stackrel{-}{{\varvec{X}}_{\varvec{e}}}$= mean score of the experimental group, $\stackrel{-}{{\varvec{X}}_{\varvec{c}}}$= mean score of the control group; ${S}_{e}^{2}$= standard deviation of the experimental group, ${S}_{\text{c}}^{2}$= standard deviation of the control group; K = ratio of the number of subjects in the experimental group to the number of subjects in the control group, K =1; ${S}^{2}$to estimate${{\upsigma }}^{2}.$ A total of 24 people were required according to the Fugl-Meyer result, and 16 people were required according to the attention network test result. Due to the possibility of sample shedding during the test, the sample size was increased by 20%, and the final number of cases included in each group was 30, amounting to a total of 60 participants in the two groups.

### Study design

This randomized controlled trial has been registered in the Chinese Clinical Trial Registry with Portal Number ChiCTR2100050430(27/08/2021) and conducted in the Department of Rehabilitation, Hebei General Hospital, from September 1,2021 to July 1,2022. Patients were randomly assigned to a MI-BCI training plus conventional rehabilitation training (BCI group) or to conventional rehabilitation training (CR group).

Inclusion criteria were the following: (1) first onset of stroke confirmed by computed tomography (CT) or magnetic resonance imaging (MRI) examination; (2) age 18–65 years, within one month of disease duration; (3) the dominant was the right and the right side was the hemiplegic side, Brunnstrom stage: upper limb for stage III-V, hand for stage II-V, elbow flexors of modified Ashworth grade ≤ 2 grade; (4) stable condition, good comprehension, able to communicate and cooperate with this study verbally; (5)score on the attention dimension of the Montreal Cognitive Assessment Scale (Moca): 2–5 points; (6) with certain motor imagery ability: the Kinesthetic and Visual Imagery Questionnaire (KVIQ-20) ≥ 55 points [22].

Exclusion criteria were the following: (1) those with severe cognitive impairment with a Mini-Mental State Examination (MMSE) score < 21 points [1], hearing impairment, visual impairment, aphasia, and other difficulties in cooperating with the training; (2) those with muscle, bone, and other neurological diseases affecting the motor function of the upper limb such as fracture of the affected upper limb, severe arthritis, joint replacement; (3) those with a clear history of other causes of cognitive impairment, such as Alzheimer’s disease, Parkinson’s disease, and other diseases; (4) those with skin damage at the contact site with electrode pads or skin damage or infection or allergy to electrodes; (5) those with intracranial metal implants, metal pacemakers or incomplete skull.

**Fig. 1:**
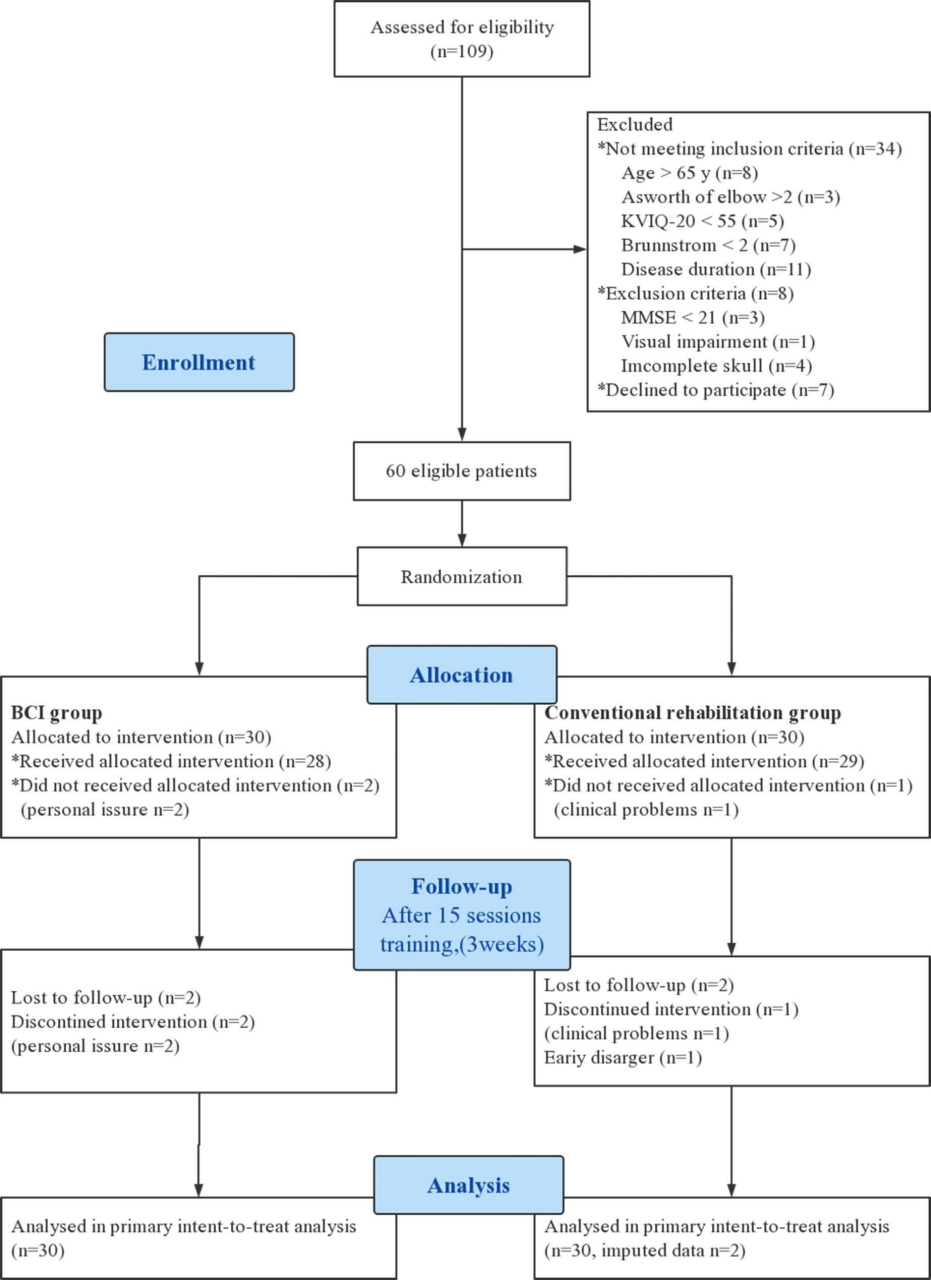
Study flowchart

Withdrawal criteria were the following: (1) patients refusing to continue to participate in the study; (2) development of an acute disease or decompensation of chronic disease with the risk of a potential impact on the study results (repeated stroke, acute myocardial infarction, non-compensated diabetes, etc.); (3) patients with 20% absence of training session. Figure 1 shows a flow chart of the trail.

### Randomization and blinding

This was a single-blind randomized clinical trial. A total of 60 subjects were randomly assigned into two groups using the envelope randomization method. Opaque envelopes of the same size were first made and encoded. The treatment regimen was determined by a random sequence generated by the computer and put in the corresponding numbered envelope. Patients were numbered according to the order of enrollment, the envelopes with the same number were opened, and the study was conducted according to the treatment plan contained in the envelopes.

In the present study, we used assessment blinding. The evaluator evaluated the findings without knowing the patient assignment, and each patient was evaluated by the same evaluator before and after the intervention to avoid changes within the scorer. Statistical analysis was carried out by persons not involved in recruitment, screening, evaluation, or intervention. In addition, the treatment process was independently performed by therapists who were not involved in information collection, evaluation, and data analysis procedures. To minimize bias and distractions, therapists did not provide patients with any information about the benefits and risks of treatment. An independent staff member oversaw the implementation of the blindfolding. If blinding failed, the patient was excluded from the study protocol.

### Interventions

Patients in both groups received conventional clinical pharmacological interventions, nursing care, and comprehensive rehabilitation treatment for hemiplegic limbs in the Department of Rehabilitation Medicine. The content of conventional rehabilitation training refer to the stroke rehabilitation guidelines [23] and included motor therapy, occupational therapy, physical factor therapy, coordination, anti-spasticity training, acupuncture, and moxibustion therapy. All training programs were performed once a day, 5 days a week, for 3 weeks. Experienced rehabilitation therapists performed all treatments, and the intensity of training was the same in both groups.

The BCI group received a 20-minute MI-BCI training in addition to the conventional rehabilitation training. The used equipment included an LSR-AII BCI rehabilitation training instrument manufactured by Shandong Haitian Intelligent Engineering Co. Patients needed motor imagery ability to use the device. Otherwise, the system could not be successfully activated [24]. KVIQ is commonly used to evaluate the motor imagery ability in stroke patients [25]. Patients with KVIQ-20 score ≥ 55points were selected for training [22]. The CR group added 20 min of functional electrical stimulation training to conventional routine rehabilitation training.

### BCI protocol

**Fig. 2:**
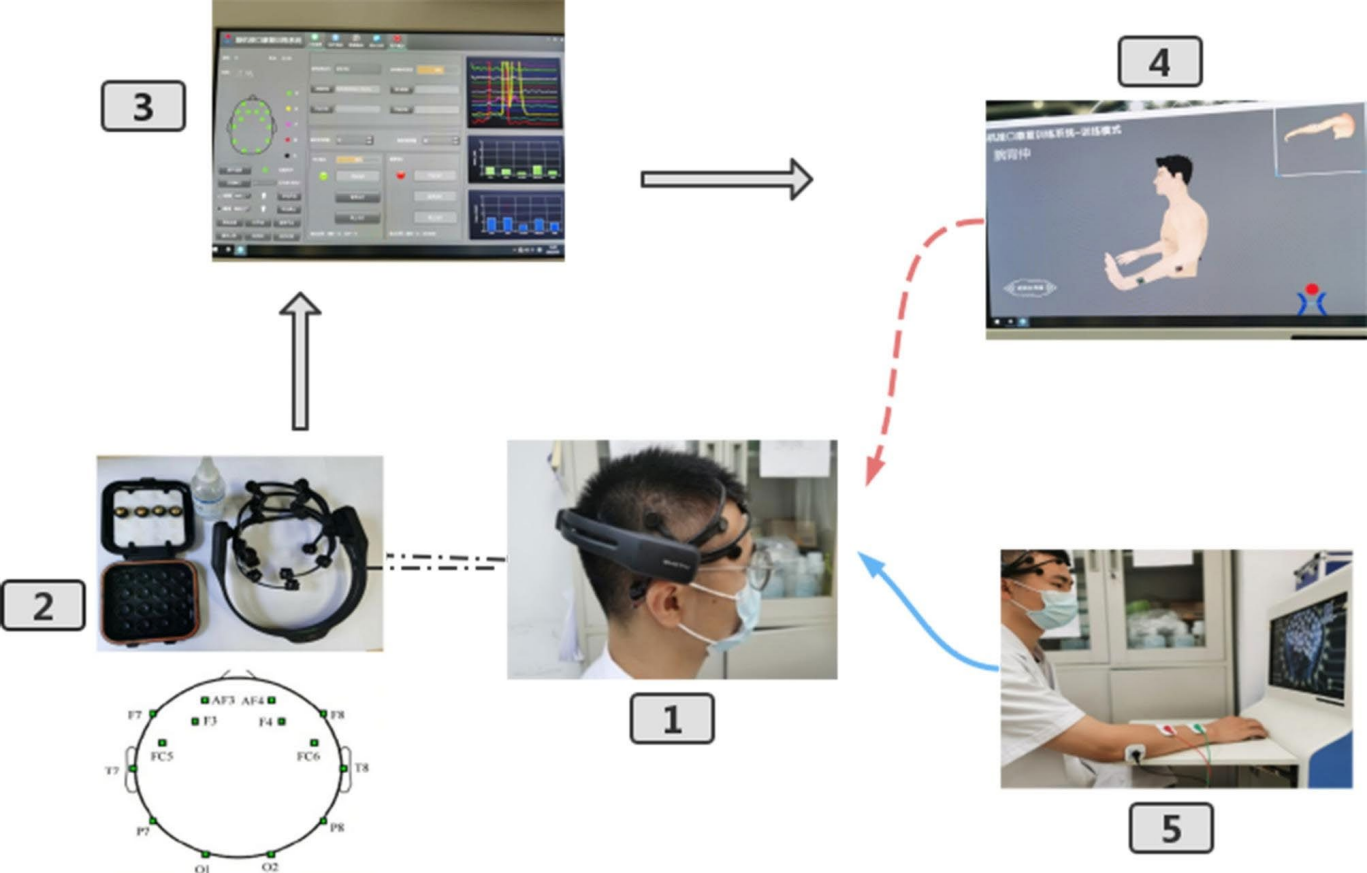
Flow chart of BCI training. (1–2) EEG amplifiers collect EEG signals generated during motor imagery. (2) Emotive Epoc + and 14 channels and two reference electrode placement positions. (3) A computer (OS Windows7): data processing and instruction conversion are performed, and motion intention is classified and decoded. (4) The computer side screen provides visual assistance and visual feedback during motor imagery. (5) The functional electrical stimulator stimulates the muscles to complete motor execution, giving sensory and perceptual feedback

The design of the BCI-controlled functional electrical stimulator is shown in Fig. 2

BCI system comprised two monitor screens (one displayed the principal computer interface for the operation of the researchers, and the other performed prompt and feedback messages), a mainframe computer to record and process brainwave activity, and a FES equipment. The treatment was carried out in an independent and quiet room and included the following treatment steps: (1) patients comfortably sat at the secondary monitor screens of the BCI system; (2) they wore the electrode caps, which were used to collect electroencephalogram (EEG) signals through the 10–20 method of international standard electroencephalogram measurement, and conductive fluid was applied to the electrodes to ensure that each electrode is in good contact according to the screen prompts. (3) The appropriate treatment action was selected according to the patient’s specific situation. Silicone electrode pads were placed on the muscles for stimulation, and the current level was slowly adjusted so that it could be tolerated by the patients [14]. (4) Patients performed MI training before formal BCI training. The patient imagined the movement with computerized voice commands and on-screen animation. The computer collected and analyzed the signals of brain activity in the imagined and calm states and calculated the degree of accuracy of the patient’s motor imagery. The process was repeated until the patient could clearly visualize the corresponding movement of the affected limb and the computer assessed the motor imagery with an accuracy of at least 60% [26]. The formal BCI training could be initiated only after the patient could manipulate the BCI. (5) During formal BCI training, motor imagery training was first performed 10 times, and the average value of the accuracy of 10 times of motor imagery was taken as the threshold of this training, and the threshold was corrected again before each formal BCI training [3]. As motor imagery varies with time, situation, and mood, the goal was to help patients achieve the best therapeutic outcomes. (6) During the training, if the attention was focused on the motor imagination process and the threshold was reached, the functional electrical stimulator was triggered to stimulate the corresponding muscle to produce the actual action and give feedback to sound vision and sensory perception, encouraging the patient to continue to focus on the BCI training. On the contrary, if the attention was not focused and the accuracy of motor imagery could not reach the threshold, only sound could help the patient correct the state and prepare for the next motor imagination. FES is triggered only when the BCI system detects a user’s intent to move, thus synchronizing the brain activity with the movement generated by muscle contraction.

The movements imagined by the exercise were: shoulder abduction, upper arm adduction, flexion of the forearm with the palm facing upward, wrist dorsiflexion, fist clenching, and wrist inversion. The selection was individualized according to the patient’s functional status and timely adjusted according to the patient’s different stages. The stimulation parameters were empirically determined to achieve the required motion without causing discomfort to the patient.

### **Evaluation** indicators

Primary and secondary outcomes were assessed at baseline and after the 3-week intervention, and the post-intervention assessment was performed one day after the last session. The primary outcome measures were the change of Fugl-Meyer Motor Function of Upper Extremity Scale and the Attention Network Test from baseline to 3 weeks. Secondary outcome measures were the change in Wolf Motor Function Test, Modified Barthel Index, Schulte Gird Test, and Symbol Digit Modalities Test from baseline to 3 weeks.

### Quantification of upper limb motor function

(1) Fugl-Meyer Motor Function of Upper Extremity Scale (FMA-UE): this is a stroke-specific performance-based impairment index that has been extensively tested, revealing to have excellent properties [27]. It is designed to assess motor functioning, sensation, and joint functioning and determine disease severity, describe motor recovery, and plan and assess treatment [28].

(2) Wolf Motor Function Test (WMFT): quantifies upper extremity movement ability through timed single- or multiple-joint motions and functional tasks. WMFT is considered an indicator of movement ability and activity, which can more sensitively measure subtle changes before and after the intervention to further support the FMA-UE evaluation from activity level [29].

(3) Modified Barthel Index (MBI): is a measure of activities of daily living (ADL), which shows the degree of independence of a patient from any assistance. It is an important method used to evaluate the capacity of participants to conduct 10 different ADLs, considered basic ADLs, thus providing a quantitative estimation of their independence level [30].

### Quantification of attention

(1) Attention Network Test (ANT): according to the attention network theory, the attention system can be divided into three anatomically and functionally independent components, i.e., alertness, orientation, and executive control. It reflects the individual’s ability to acquire and maintain a state of alertness to a certain type of information, selective attention to use external information, and processing conflicting information [31]. The Attention Network Test (ANT), designed by Fan et al. [32], records three networks’ positive and negative responses and response time (RT) by computer. ANT provides a useful tool for investigating the alert, orientation and executive control functions of the attention network [31]. This reliable tool has been widely used in the assessment of attention disorders. It can also be used to detect occult attention deficits in stroke patients [33]. The longer alertness network RT and orientation network RT are preferred. Also, shorter executive control network RT, total average RT, and total time are preferred.

(2) Schulte Grid Test: is a classic attention assessment and training tool [34], where a software program on the user side of the cell phone is used to assess the subjects. During the test, the distance between the square and the eye is required to be 35–40 cm, clear and fully visible, and the point of view is placed in the center of the table, and the subject presses the positions 1–25 in turn with his hand while reciting the sound, and the software records the time spent at the end. The evaluation index was the time needed to count all 25 digits. In order to improve the test reliability, the average level of the five tests was taken to represent the attention level of the subjects. The shorter the time needed to count the digits indicated, the higher level of attention. The test was explained to the patients before the initial assessment and was also practiced before the assessment using 3 × 3 and 4 × 4 forms. The test was given after the method was fully mastered and when the patient was emotionally stable and in good condition.

(3) Symbol Digit Modalities Test (SDMT): subjects were asked to fill in the number of the corresponding character in the box corresponding to the symbol below as soon as possible, according to the symbol at the top of the page and its corresponding number. The number of items correctly completed within 90 s was scored on the scale. Changes in scores 3–4 were considered clinically relevant [35]. SDMT can be used to assess information processing speed in stroke patients with high reliability and test-retest reliability. In particular, the verbal version of the SDMT sidesteps the issues associated with the lack of ability to perform paper-and-pencil tests due to motor deficits [7].

### Statistical analysis

Exploratory data analysis and Shapiro-Wilk test were performed to determine the normality of the data distribution. Continuous variables were expressed as means (SD) or medians (Q_1_, Q_3_). Mean differences were expressed with their 2-sided 95% CIs. Between-group differences at baseline and in the change from baseline to the end of the study were tested with unpaired the Mann-Whitney U test. Gender, Brunnstrom staging, and modified Ashworth grading data, which were categorical variables, were expressed as counts and percentages. Between-group comparisons at baseline in categorical variables were tested with the Chi-square test. The Spearman correlation test was used if one of the variables did not conform to the normal distribution. A *P* < 0.05 represented statistical significance. All analyses were conducted with SPSS version 25.0 statistical software (IBM Inc., Chicago, IL, USA).

## Results

The study flow chart is shown in Fig. 1. A total of 109 patients were diagnosed with stroke. Of those, 60 were deemed eligible. Two patients in the BCI group were excluded due to personal reasons (gave up MI-BCI training and continued conventional rehabilitation training, n = 2) and two patients were excluded from the conventional rehabilitation group (one due to atrial fibrillation and one due to early discharge on account of unexpected personal reasons, n = 2). The baseline characteristics of the participants are shown in Table 1. There were no significant baseline differences in the two groups in terms of sex, age, duration of disease, stroke type, years of education, Brunnstrom classification of upper limb and hand, Ashworth rating of elbow flexor, and KVIQ-20 score (all *P* > 0.05). Furthermore, there were no differences between the groups in outcome measures such as the FMA-UE, ANT, WMFT, MBI, Schulte Grid test, and SDMT before the intervention(Mann-Whitney U test, all *P* > 0.05), as shown in Tables 2 and 3.

**Table 1:** Comparison of clinical characteristics between two groups

Characteristics	BCI group (n = 30)	CR group (n = 30)	*P*-value
Sex			
Male	22(73.3)	19(63.3)	0.629
Female	8(26.7)	11(36.7)	
Age (years)	52.5(45.0, 59.3)	53.0(38.5, 59.5)	0.599
Duration of disease (days)	18.5(12.0, 24.3)	18.0(9.8, 23.0)	0.496
Stroke Type			
Cerebral infarction	22(73.3)	17(56.7)	0.176
Cerebral hemorrhage	8(26.7)	13(43.3)	
Years of education (years)	9.0(6.0, 14.3)	9.0(6.0, 10.3)	0.234
Brunnstrom classification of upper limb			
III	16(55.3)	14(46.7)	0.837
IV	6(20.0)	6(20.0)	
V	8(26.7)	10(33.3)	
Brunnstrom classification of hand			
II	5(16.7)	2(6.7)	0.730
III	11(36.7)	13(43.3)	
IV	6(20.0)	7(23.3)	
V	8(26.7)	8(26.7)	
Ashworth rating of elbow flexor			
0	17(56.7)	14(46.7)	0.690
1	9(31.0)	10(33.3)	
2	4(13.3)	6(20.0)	
KVIQ-20 score	65.0(60.0, 72.0)	62.5(58.8, 65.5)	0.166

NOTE! Values are n (%) or Medians, and 25 and 75% quartiles are shown; BCI: brain-computer interface; CR: conventional rehabilitation; KVIQ-20: the kinesthetic and visual imagery questionnaire

### **Motor** improvements

The results of the motor assessment are shown in Table 2. After 3 weeks of training, the change in FMA-UE was significantly different between groups by 8.0 points (95% CI, 5.0 to 10.0; *P* < 0.001), with a median (Q_1_, Q_3_) change of 11.0 (8.0, 16.3) points in the BCI group vs. 4.0 (2.8, 5.0) points in the conventional rehabilitation group. Similarly, after 3 weeks of training, WMFT (8.0 points;95% CI, 6.0 to 11.0; *P* < 0.001) and MBI (17.0 points; 95% CI, 12.0 to 21.0; *P* < 0.001) were significantly increased in the BCI group compared with the conventional rehabilitation group.

**Table 2:** Comparison of FMA-UE, WMFT, and MBI points between two groups

Variables		BCI group (n = 30)	CR group (n = 30)	Estimated Difference BCI vs. CR (95%CI)	*P-*value
FMA-UE	Baseline	29.0(21.8, 42.3)	36.0(24.0, 43.3)	−2.5 (− 10.0 to 3.0)	0.351
	Final	46.0(33.8, 55.8)	39.5(27.8, 45.0)	6.0 (0.0 to 13.0)	0.049
	Change-value	11.0(8.0, 16.3)	4.0(2.8, 5.0)	8.0 (5.0 to 10.0)	< 0.001
WMFT	Baseline	30.5(22.0, 43.8)	31.5(24.8, 45.5)	−2.0 (− 9.0 to 4.0)	0.433
	Final	51.5(38.0, 59.8)	40.0(32.8, 50.3)	9.0 (1.0 to 17.0)	0.033
	Change-value	13.5(9.8, 22.3)	5.0(3.8, 6.3)	8.0 (6.0 to 11.0)	< 0.001
MBI	Baseline	54.5(45.0, 65.8)	52.5(43.8, 68.5)	0.0 (− 7.0 to 7.0)	0.965
	Final	80.0(70.0, 93.3)	62.5(48.8, 72.2)	18.0 (9.1 to 25.0)	< 0.001
	Change-value	24.0(16.8, 30.0)	6.5(4.0, 9.0)	17.0 (12.0 to 21.0)	< 0.001

Medians and 25 and 75% quartiles are shown; Change-value:final-baseline; BCI: brain-computer interface; CR: conventional rehabilitation; FMA-UE: Fugl-Meyer Motor Function of Upper Extremity Scale; WMFT: Wolf Motor Function Test; MBI: Modified Barthel Index

### **Attention** improvements

The results of the attention assessment are shown in Table 3. After 3 weeks of training, alert network response time (32.4ms; 95% CI, 58.4 to 85.6; *P* < 0.001) and orienting network response time (5.6ms; 95% CI, 29.8 to 55.8; *P* = 0.010) as well as corrects number (8.0; 95% CI, 17.0 to 28.0; *P* < 0.001) were significantly increased in the BCI group compared with the conventional rehabilitation group. Individuals in the BCI group reduced executive control response time with a median (Q_1_, Q_3_) change of − 51.8ms (− 96.9, − 12.3), *P =* 0.001, while it remained unchanged in those in the conventional rehabilitation group 4.7ms (− 19.3, 49.6) *P =* 0.428, resulting in a between-group difference of − 105.9ms (95% CI, − 68.3 to 23.6; *P* = 0 0.002). Total average response time (− 244.8ms; 95% CI, − 155.8 to − 66.2; *P* = 0.002) and total time (− 122.0s; 95% CI, − 80.0 to − 35.0; *P* = 0.001) were significantly reduced in the BCI group compared with the conventional rehabilitation group. After 3 weeks of training, there were also significant between-group differences in the change in SDMT (8.0; 95% CI, 6.0 to 9.0; *P* < 0.001) and Schulte Gird test (− 19.7s; 95% CI, − 12.8 to − 8.9; *P* < 0.001) in favor of the BCI group.

**Table 3:** Comparison of ANT, Schulte Gird test, and SDMTV between two groups of patients

Variables	Group	Baseline	Final	Change-value	ED, (95%CI)	*P-*value
ANT Alert network RT(ms)	BCI	90.6(47.0, 135.5)	117.9(81.0, 172.7)	31.7(10.5, 60.5)	32.4, (58.4 to 85.6)	< 0.001
	CR	97.6(51.0, 178.6)	76.26(35.2, 120.6)	−20.6(− 69.0, 26.9)		
Orienting network RT(ms)	BCI	30.2(11.2, 47.7)	50.5(28.1, 98.7)	17.86(1.5, 53.0)	5.6, (29.8 to 55.8)	0.010
	CR	45.8(14.8, 94.6)	51.5(23.1, 68.4)	−1.67(− 38.5, 24.4)		
Executive control network RT(ms)	BCI	182.9(136.3, 220.9)	130.0(90.0, 182.7)	−51.8(− 96.9, − 12.3)	−105.9, (− 68.3 to − 23.6)	0.002
	CR	135.7(105.8, 186.8)	156.3(124.2, 198.4)	4.7(− 19.3, 49.6)		
Total average RT(ms)	BCI	1106.4(984.4, 1353.3)	956.5(847.4, 1127.3)	−121.7(− 260.1, − 54.2)	−244.8, (− 155.8 to − 66.2)	0.002
	CR	1194.6(970.3, 1452.3)	1204.2(1023.8, 1464.1)	10.5(− 131.0, 136.6)		
Total time(s)	BCI	880.0(848.5, 973.3)	832.0(797.5, 890.5)	−44.5(− 110.5, − 17.0)	−122.0, (− 80.0 to − 35.0)	0.001
	CR	922.0(836.2, 1031.0)	936.0(883.5, 1057.0)	28.0(− 53.3, 78.8)		
Corrects number	BCI	286.5(266.0, 307.0)	308.5(299.5, 310.3)	15.5(3.0, 41.3)	8.0, (17.0 to 28.0)	< 0.001
	CR	299.5(254.0, 307.3)	291.9(269.8, 300.0)	0.0(− 9.3, 18.3)		
SDMT	BCI	25.0(20.8, 34.0)	35.0(29.8, 41.5)	8.5(6.0, 11.3)	8.0, (6.0 to − 9.0)	< 0.001
	CR	28.5(24, 32.3)	29.5(25.0, 34.0)	2.0(− 0.25, 2.0)		
Schulte Gird test(s)	BCI	56.8(48.8, 87.1)	44.4(35.9, 56.8)	−14.3(− 29.8, − 9.6)	−19.7, (− 12.8 to − 8.9)	< 0.001
	CR	56.7(43.8, 68.5)	56.8(45.2, 70.0)	−3.2(− 5.4, 1.6)		

Medians and 25 and 75% quartiles are shown; Change-value:final-baseline; RT:response time; BCI: brain-computer interface; CR: conventional rehabilitation; ANT: Attention Network Test; SDMT: Symbol Digit Modalities Test; ED:estimated difference

### Correlation analysis of attention improvement and motor function improvement

**Fig. 3:**
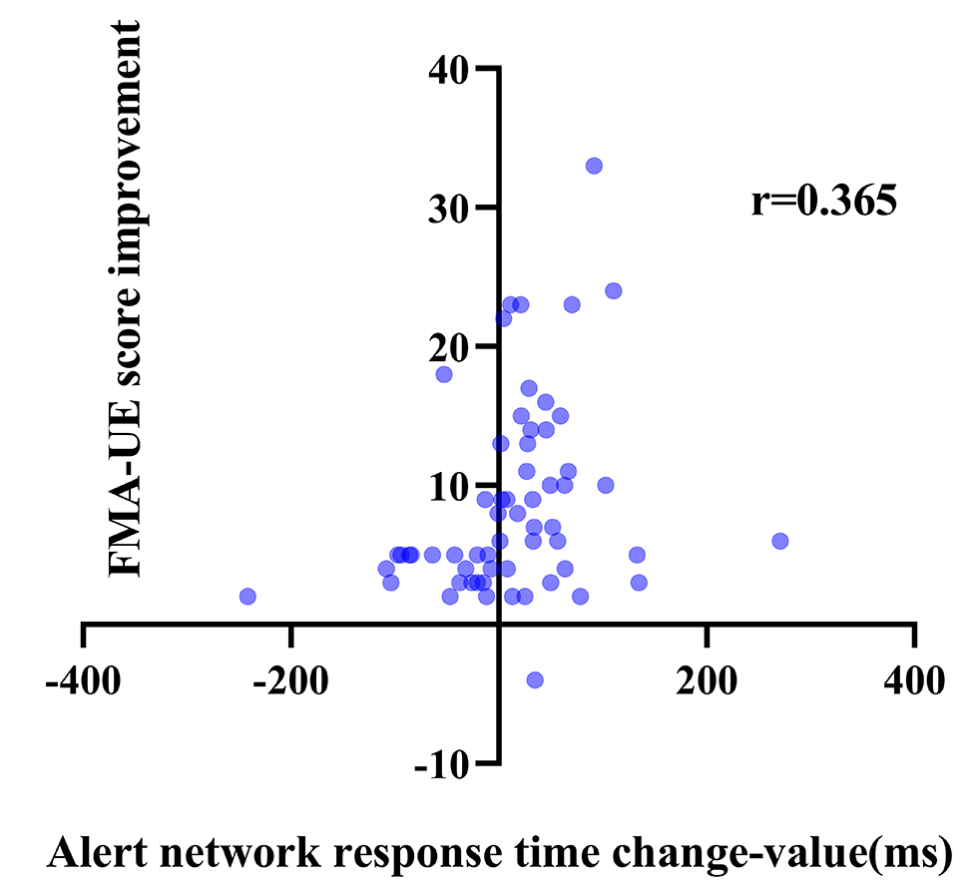
Correlation analysis between the change in alert network response time and the FMA-UE score improvement

**Fig. 4:**
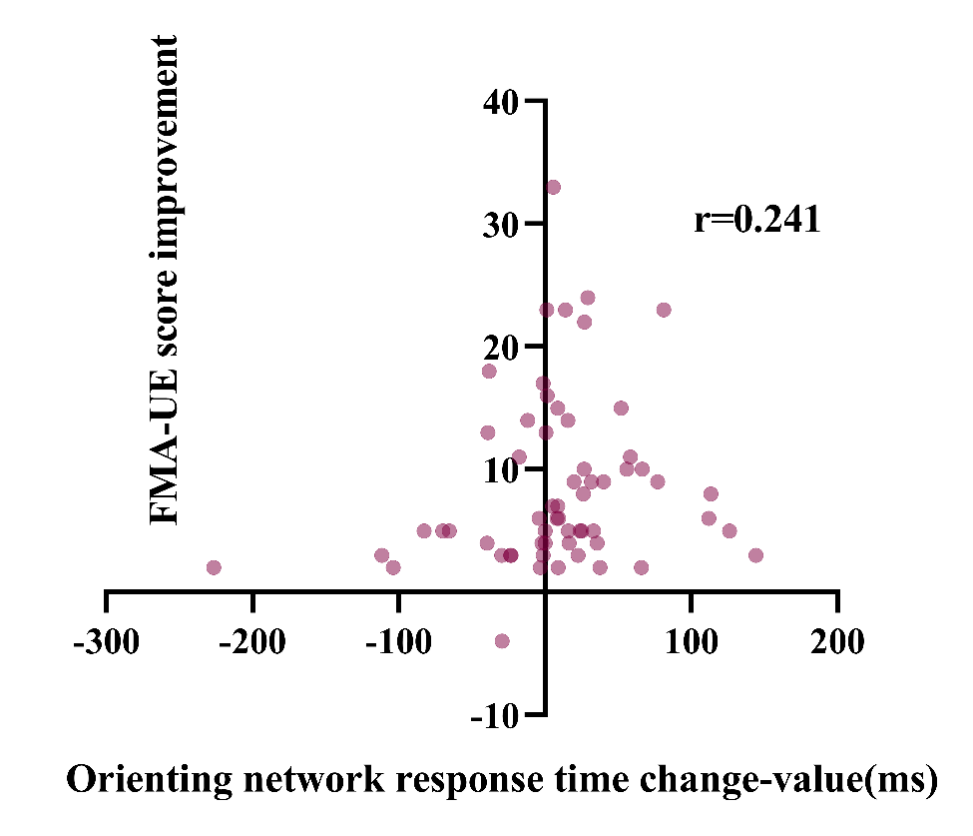
Correlation analysis between the change in orienting network response time and the FMA-UE score improvement

**Fig. 5:**
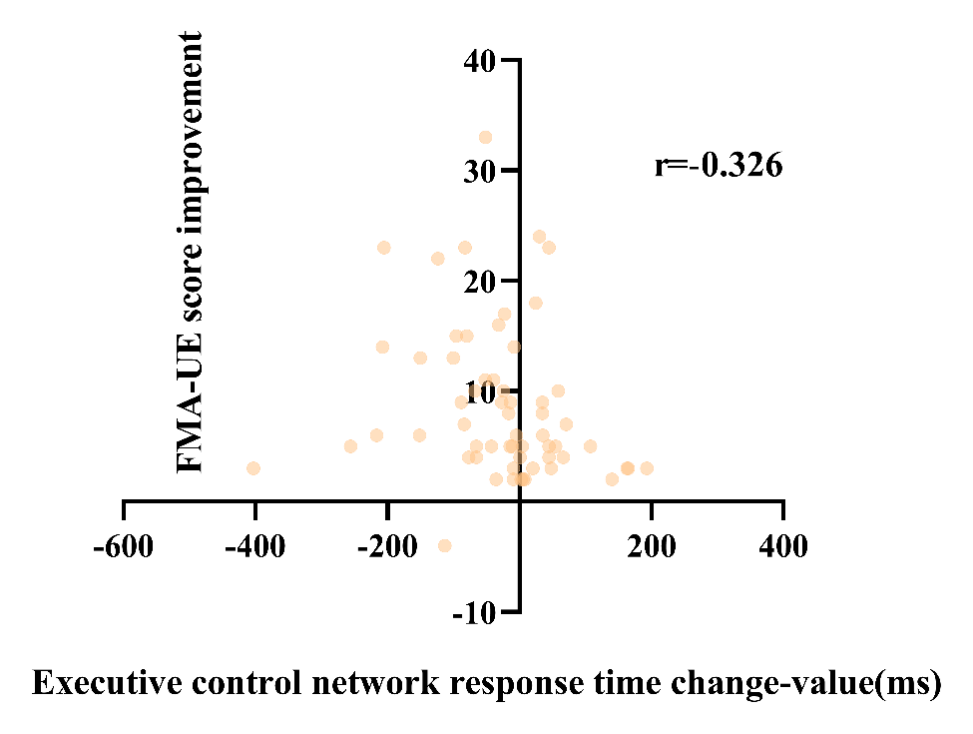
Correlation analysis between the change in executive control network response time and the FMA-UE score improvement

**Fig. 6:**
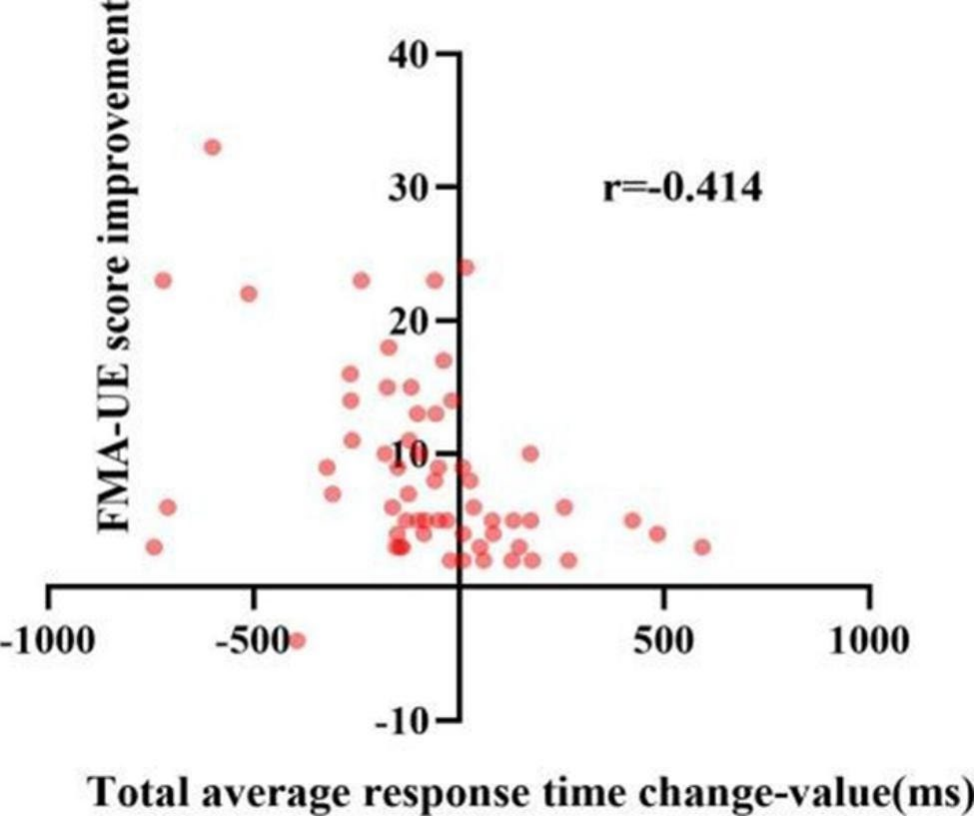
Correlation analysis between the change in total average response time and the FMA-UE score improvement

**Fig. 7:**
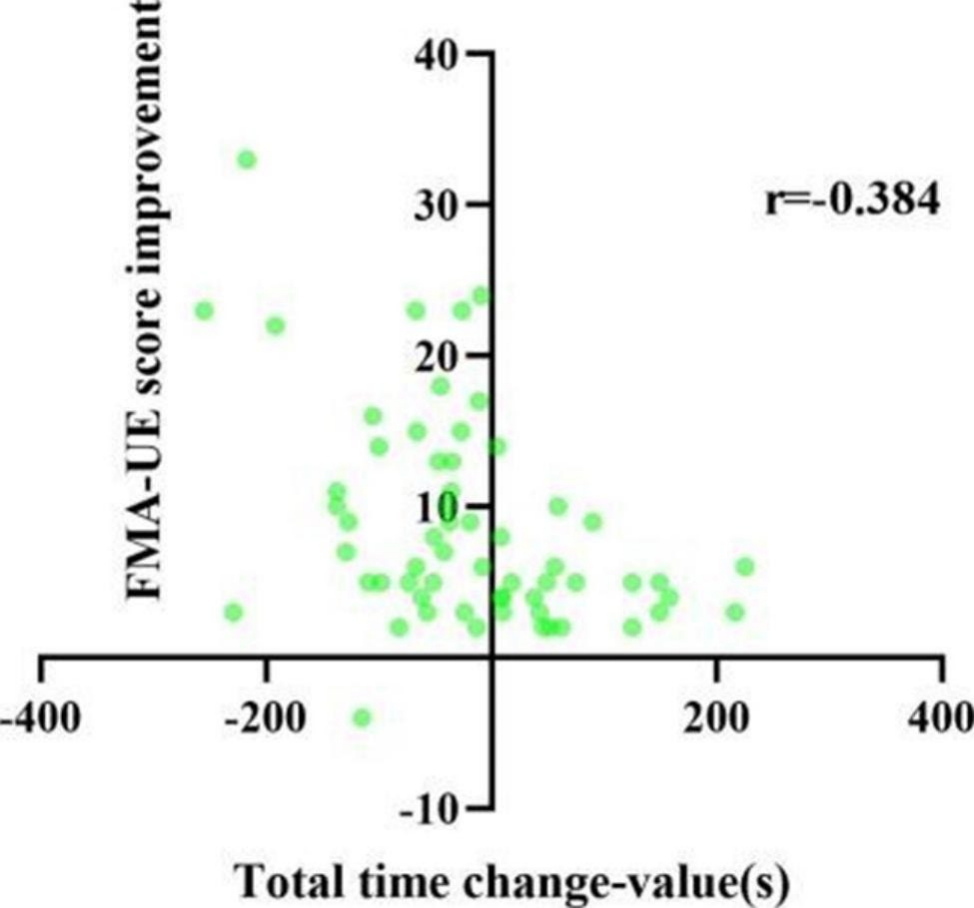
Correlation analysis between the change in total time and the FMA-UE score improvement

**Fig. 8:**
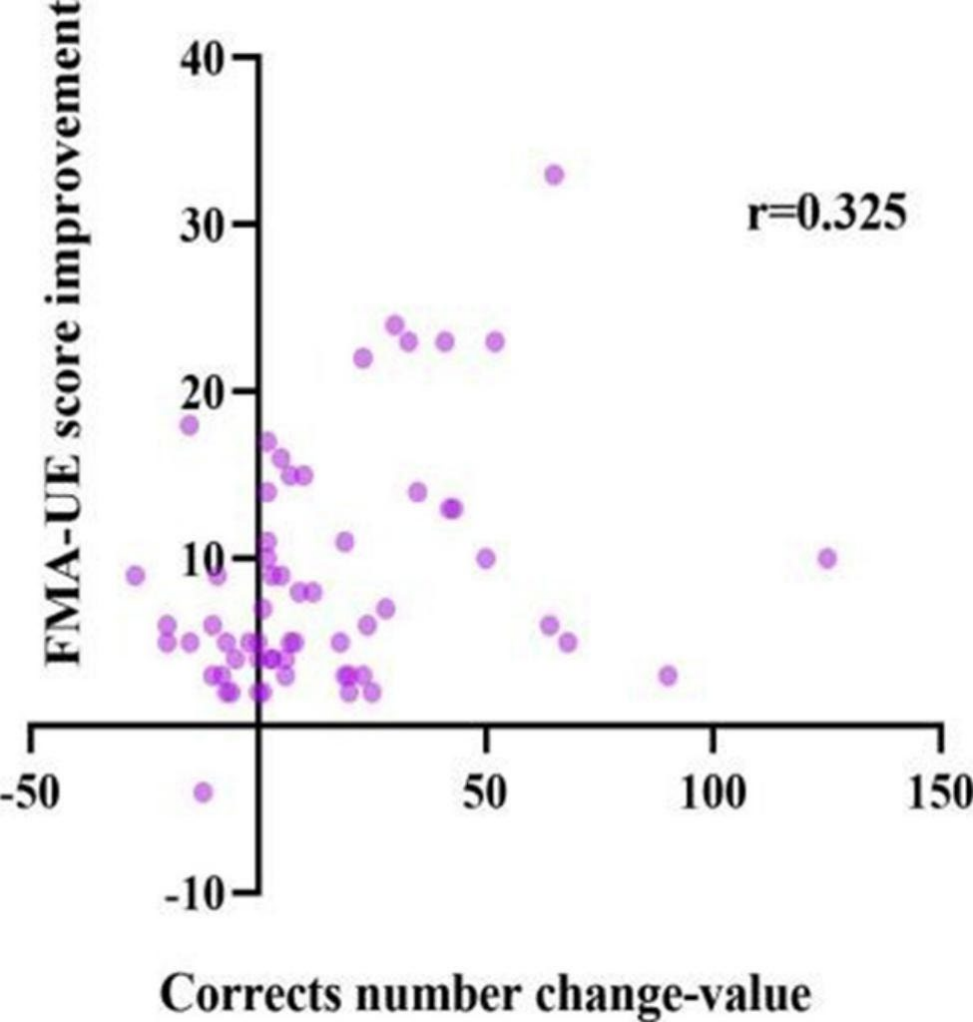
Correlation analysis between the change in corrects number and the FMA-UE score improvement

To further investigate the relationship between attention and motor function, we analyzed the correlation between attention improvement and motor function recovery. The results of the correlation analysis of attention improvement and motor function improvement are shown in Table 4. Overall, after 3 weeks of training, results showed the correlation was significant for improvement in motor function and attention improvement. Only the change in orienting network response time with FMA-UE score improvement and the change in the correct number with WMFT score improvement were weakly correlated (*P* > 0.05). Figures 3, 4, 5, 6, 7 and 8 shows the correlation analysis between the improvement in the main motor function outcome index (FMA-UE) and the change in each item of the main attention outcome index (ANT). The improvement of the FMA-UE score was positively correlated with the changes in alert network response time and correct number (*P* < 0.05). The change in orienting network response time with the improvement of the FMA-UE score was not correlated (*P* > 0.05). A negative correlation was found between the change in executive control network response time and FMA-UE score improvement (*P* < 0.001). Changes in total average response time and total time were also negatively correlated with improvements in FMA-UE scores (*P* < 0.001).

**Table 4:** Correlation analysis of the improvement of motor function and the improvement of attention level (r)

Variables (Change-value)	FMA-UE (Change-value)	WMFT (Change-value)	MBI (Change-value)
SDMT	0.693^**^	0.662^**^	0.733^**^
Schulte Gird test(s)	−0.493^**^	0.505^**^	0.631^**^
Alert network RT(ms)	0.365^**^	0.379^**^	0.446^**^
Orienting network RT (ms)	0.241	0.310^*^	0.294^*^
Executive control network RT(ms)	−0.326^*^	−0.267^*^	−0.290^*^
Total average RT(ms)	−0.414^**^	−0.314^*^	−0.350^**^
Total time(s)	−0.384^**^	−0.259^*^	−0.432^**^
Corrects number	0.325^*^	0.247	0.431^**^

^*^P < 0.05 ^**^P < 0.01; SDMT:Symbol Digit Modalities Test; RT: response time; FMA-UE: Fugl-Meyer Motor Function of Upper Extremity Scale; WMFT: Wolf Motor Function Test; MBI: Modified Barthel Index

## Discussion

In the present study, we introduced the results of a clinical study on the effects of MI-BCI combined with conventional rehabilitation training on upper limb function and attention in stroke patients with hemiplegia and compared these results with those of conventional rehabilitation training. The correlation between motor function recovery and attention improvement was also explored.

Improvements in motor function were reflected by the changes in FMA-UE, WMFT, and MBI from the baseline to 3 weeks. Our results showed a significant improvement in motor function in the BCI group compared with the conventional rehabilitation group. The results of our study are consistent with the results of the previous controlled studies that employed motor-imagery BCIs. In particular, Long [36] et al. reported a controlled trial with 32 chronic post-stroke patients: 16 patients trained with a motor imagery-based BCI with functional electrical stimulation feedback (BCI-FES) coupled with physiotherapy, and 16 patients received neuromuscular electrical stimulation (NMES) and physiotherapy alone. For the BCI-FES group, each training session lasted for 40 min, with a total of 11 sessions. An improvement of the motor function in the BCI-FES group, as assessed with FMA-UE, was significantly greater than that in the NMES group. In a study by Biasiucci et al. [37] which involved 30 chronic stroke patients, the treatment effect was compared between a group of subjects who received training with BCI-controlled FES and a group of subjects treated only with the sham-FES. Both groups received treatment for about one hour twice a week for 5 weeks. In this case, motor function was significantly improved in the BCI-FES group compared with the sham-FES group, and the improvement persisted for at least 6 months. In addition, Ang [13] et al. presented the results of a randomized controlled trial of 11 patients with chronic stroke who received an MI-BCI system with shoulder-elbow robotic feedback, compared with 15 who received shoulder-elbow robotic feedback not linked to MI-BCI. On the basis of regular rehabilitation, both groups received a total of 18 h of intervention, which were delivered over 4 weeks. Patients who received BCI feedback showed significantly greater motor improvements, measured by FMMA scores. It should be noted that, in contrast to our study, patients in the aforementioned studies received much greater training intensity than our study, where the overall training time was five hours. However, in spite of the short training time we obtained significant differences between all results in motor function. MI-BCI integrating both BCI and MI central interventions can better exploit their synergistic effects and significantly improve patients’ motor function and activities of daily living [10]. At the same time, MI-BCI integrated with FES couples real-time motor intention with motor execution, which continuously repeats the reinforcement learning process, thus improving the motor relearning ability of patients and promoting the recovery of impaired limb motor function.

This study also explored the changes in the ANT, SDMT, and Schulte Grid test as indexes to assess the improvement of attention in stroke patients. An increase in the alert and orienting network response time and the correct number, as well as a decrease in the executive control network and total average response time and total time, were observed, suggesting an improvement in attention. Results showed that participants in the BCI group obtained greater improvements in attention than the participants in the conventional rehabilitation group. MI-BCI could improve the information processing speed and alertness to the outside world in stroke patients, thus improving their attention level, which is also consistent with previous studies. Kim and Lee [38] conducted BCI-FES training for 30 times on 9 patients with spastic cerebral palsy, during which patients were required to concentrate on triggering FES. Their results showed that BCI could not only improve motor function but could also improve attention level, increase logical thinking and affect functional brain activity. Gomez-Pilar et al. [39] used MI-BCI to train motor imagery combined with logical memory tasks five times in the elderly, finding that neurofeedback training performed by MI-BCI could enhance cognitive ability. The BCI system can provide neurofeedback interventions to help patients regulate their brain state in real-time, thus improving cognitive function and attentional state [12]. Also, continuously repeating and reinforcing this process allows patients to improve their attention level. These results suggest that MI-BCI could be used as an intervention for attention disorders and can significantly improve the attention level of stroke patients.

Our results also indicated that enhanced sustained attention and information processing speed, as assessed by ANT, SDMT, and Schulte Grid test, were positively correlated with improved motor function levels. This is similar to previous studies that have found the BCI system can influence overall rehabilitation outcomes in terms of motor and cognitive aspects [12, 40]. Ortiz et al. [41] demonstrated that BCI could enhance neuroplasticity, promote cognitive and motor rehabilitation, improve the operational accuracy of exoskeletal control, and improve the attention level during walking. Salvadori et al. [42] performed attention training on attention deficit stroke patients, finding that attention improvements could help patients improve their daily living ability and balance. Individuals with efficient attentional networks performed better in motor control. Contrary to our findings, Mullick et al. [18] found only a weak association between attention and increased arm impairments and activity limitations. However, the reason for the difference may be that the stage and degree of stroke population selected in the present study are different from those selected in the meta-analysis. The nature of the correlation between cognitive domains and motor function recovery may differ between the acute and chronic phases of stroke. This correlation encourages further in-depth exploration of the mutual facilitation of motor function and attention. Using the mutual promotion of motor function recovery and attention improvement, individualized rehabilitation programs can be developed to help stroke patients gain more benefits.

The MI-BCI system can transform the accuracy of MI into quantifiable metrics and provide real-time feedback to patients. Feedback comes in the form of visual, auditory, haptic and kinesthetic feedback [36]. Indeed, increasing sensory input and output during exercise training has been shown to drive brain plasticity. This was especially evident in sensorimotor areas following somatosensory stimulation [37]. Visual feedback is the direct observation of the entire process of limb movement. Foong et al. [43] found that MI-BCI employing only visual feedback could also help stroke survivors sustain short-term motor function improvement. Audible feedback used to encourage the patient or remind the patient to correct an incorrect MI state based on the degree of accuracy of the MI. Haptic and kinesthetic feedback, on the other hand, is the sensory feedback delivered to the brain by the FES [44]. In a small pilot trial, Ono et al. [45] demonstrated that visual-kinesthetic feedback provides benefits compared to pure visual feedback for motor imagery-based BCI training in post-stroke subjects. Moreover, several BCI studies involving this type of haptic and kinesthetic feedback have demonstrated improvements in clinical parameters of post-stroke motor recovery [14, 37, 46]. These processes promote the formation of the “central-peripheral-central” stimulation circuit and accelerate the recovery of motor function. “Central-peripheral-central” is a closed-loop rehabilitation technique, where “closed-loop” refers to the complex brain feedback loops and sensorimotor interactions between the brain and environment [47]. During MI-BCI training, patients required to actively issue instructions in the brain to activate the corresponding brain areas and circuits that promote neural remodeling, coupled with the ongoing cycles of sensory and motor processing constitute a closed-loop feedback system continuously feeding sensory information to the central nervous system and reinforcing the correct motor patterns.

The closed-loop BCI system can also be used as a neurofeedback platform to improve and enhance individual cognitive performance [48]. Patients can self-regulate their attention to MI tasks based on the feedback results, thus improving their attention level. It is difficult but feasible to adjust the BCI threshold according to the motor imagination’s accuracy during each treatment for each patient. This threshold setting can help the patient actively participate in the training and maintain a high level of attention to complete the motor imagery task. It has an important role in promoting brain plasticity. Because of the two-way interaction between the brain and the computer, the MI-BCI system is used to alter brain function in stroke patients through neuroplasticity, the process of reorganization in the brain [4]. Brain plasticity can be better activated through neurofeedback-based learning [49]. Cognitive training using BCI can improve the clinical outcomes of patients and improve the availability of BCI [40]. Currently, rehabilitative BCI training systems are gradually being applied to stroke patients to activate brain plasticity, promote the recovery of neural networks and strengthen functional connectivity between brain regions, aiming to improve their motor and cognitive functions [50].

### Study limitations

The present study has some limitations. First, the medium- and long-term efficacy of MI-BCI have not been analyzed. Also, quantitative measures such as functional magnetic resonance imaging were not combined with behavioral and psychological studies to better demonstrate the effects of training. Second, this was a single-center study with a small sample size. Future multicenter studies with bigger sample sizes are needed to further verify reported findings.

## Conclusion

MI-BCI can be used to identify brain activity, classify and extract information, decode subjects’ motor intentions, and promote interneuronal interactions in combination with FES. Furthermore, MI-BCI system can be personalized to the patient’s exercise level and brain state so as to obtain better rehabilitation results. With the development of artificial intelligence technology and rehabilitation medicine, MI-BCI is gradually becoming a new neurorehabilitation treatment tool, which has improved therapeutic effect on upper limb function and attention rehabilitation in stroke patients in combination with conventional rehabilitation methods and can effectively improve treatment efficiency, and shorten the treatment period. Therefore, this training method may be feasible and suitable for stroke patients and can also gain wider application in a clinical setting.

## Data Availability

The datasets used and/or analyzed during the current study are available from the corresponding authors on reasonable request.

## Abbreviations

MI: Motor imagery
BCI: Brain-computer interface
MI-BCI: Motor imagery-based brain-computer interface
CR: Conventional rehabilitation
FMA-UE: Fugl-Meyer Motor Function Assessment of Upper Extremities
ANT: Attention Network Test
FES: Functional electrical stimulators
ADHD: Attention deficit hyperactivity disorder
SMR: Sensory-motor rhythm
CT: Computed tomography
MRI: Magnetic resonance imaging
Moca: Montreal Cognitive Assessment Scale
KVIQ-20: Kinesthetic and Visual Imagery Questionnaire
MMSE: Mini-Mental State Examination
EEG: Electroencephalogram
OS: Operating system
WMFT: Wolf Motor Function Test
MBI: Modified Barthel Index
RT: Response time
SDMT: Symbol Digit Modalities Test
NMES: Neuromuscular electrical stimulation
ED: Estimated difference.
